# Benign Orbital Tumors with Bone Destruction in Children

**DOI:** 10.1371/journal.pone.0032111

**Published:** 2012-02-24

**Authors:** Jianhua Yan, Sheng Zhou, Yongping Li

**Affiliations:** The State Key Laboratory of Ophthalmology, Zhongshan Ophthalmic Center, Sun Yat-sen University, Guangzhou, Guangdong Province, The People's Republic of China; Faculty of Medicine University of Leipzig, Germany

## Abstract

**Purpose:**

To present rare benign orbital tumors with bone destruction in children who could not be diagnosed pre-surgically and may simulate malignant ones.

**Methods:**

A retrospective review of cases. Clinical, operative and pathological records in all children with a diagnosis of benign orbital tumors who showed remarkable bone destruction at a tertiary Ophthalmic Center in China between Jan 1, 2000 and Dec 31, 2009 were reviewed. All patients had definitive histopathologic diagnosis.

**Results:**

Eight patients with benign orbital tumors showed obvious bone destruction, including six cases of eosinophilic granuloma, one case of leiomyoma and one case of primary orbital intraosseous hemangioma. Among them, three patients were females and five patients were males. Tumors were unilateral in all cases, with both the right and left side affected equally. Age ranged from 3 to 7 years (mean 4.1 years). Symptom duration ranged from 1 to 5 weeks (mean 4.8 weeks). Eyelid swelling and palpable mass were the most common complaint. There was no evidence for multifocal involvement in cases with eosinophilic granuloma. Among six patients with eosinophilic granuloma, two were treated with low dose radiation (10 Gy), three received systemic corticosteroid and one was periodically observed only after incisional biopsy or subtotal curettage. There was no postoperative therapeutic intervention in the two patients with leiomyoma and intraosseous hemangioma. All eight patients regained normal vision without local recurrence after a mean follow-up time of 32.8 months.

**Conclusion:**

Benign orbital tumors such as isolated eosinophilic granuloma, leiomyoma and primary orbital intraosseous hemangioma may show remarkable bone destruction.

## Introduction

In childhood, a large number of lesions can potentially cause bone destruction of the bony orbit with or without a solid soft tissue component. The most common benign tumor is the dermoid inclusion cyst, others such as fibrous dysplasia, juvenile ossifying fibroma are relatively common too [1]. All of these benign masses have some distinctive clinical features and can be easily diagnosed pre-surgically in clinical practice. Rhabdomyosarcoma, myelogenous leukemia, neuroblastoma and osteosarcoma are the common malignant lesions which may demonstrate bone destruction of the orbit in children [1], [2], [3]. However, some benign tumors with orbital bone destruction, though uncommon, could not be diagnosed pre-surgically and may simulate malignant ones, which can strongly bias doctor's decision-making in dealing with the disease. Here, authors present their patients who show remarkable bone destruction with a diagnosis of benign orbital tumors.

## Methods

Authors performed a retrospective review of patients with a diagnosis of benign orbital tumors which showed remarkable bone destruction in computed tomography scan and were treated at Zhongshan Ophthalmic Center, Sun Yat-sen University, Guangzhou, China between Jan 1, 2000 and Dec 31, 2009. The ethics committee of Zhongshan Ophthalmic Center approved this retrospective study and our paper has been conducted according to the principles expressed in the Declaration of Helsinki. The committee specifically waived the need for consent. The persons concerned (or their legal guardians) have seen this manuscript and figures and have provided written consent for publication. The clinical, operative and pathological records were reviewed, and orbital CT scans were examined. All patients had definitive histopathologic diagnosis, with all histologic patterns reexamined by two observers without knowledge of the previous diagnosis and clinical outcome. Among the inclusion criteria were that all patients had treatment by a single surgeon (JH Yan); systemic evaluation by a pediatric oncologist, including a complete medical history and physical examination, laboratory studies, and a bone scan; and minimum follow-up of 12 months.

The data collected in this study included the general data such as the patient's age, sex, the duration of orbital lesion at presentation. The ocular data included the affected orbit, laterality, symptoms (visual problem, red or swelling, proptosis, diplopia, palpable mass), signs (the best corrected vision, proptosis, ocular motility deficit, strabismus). Tumor data included orbital location (superior, inferior, anterior, posterior), configuration (round, ovoid, diffuse), size, margin (ill-defined, well-defined), quality (rigid, soft, medium), tenderness (present, absent), tissues or spaces involved, imaging findings, histopathologic examination.

All patients had a presumed diagnosis of orbital malignancy before surgery. Usually, initial management involved incisional biopsy or subtotal curettage. This generally was accomplished through anterior orbitotomy. Tissue was submitted for pathological evaluation. After pathological diagnosis, the postoperative treatment included close follow-up observation, low-dose irradiation, or systemic corticosteroid.

## Results

Eight patients met the inclusion criteria for this study (Table 1). Among them, 5 patients were female and 5 male and none had a previous history of serious illness. Involvement was unilateral in all cases, with both the right and left side affected in four cases. Age at time of first diagnosis ranged from 3 to 7 years (mean 4.1 years). Symptom duration ranged from 1 to 5 weeks (mean 4.8 weeks). Eyelid swelling and palpable mass was the most common complaint; four of eight patients reported pain or tenderness. Vision was mildly decreased in three patients. All patients underwent incisional biopsy or subtotal curettage. However, the curettage of grossly abnormal tissue was limited to the orbital components, and abnormal tissue was not pursued into the epidural space, temporal fossa, or forehead.

**Table 1:** The clinical data in 8 cases of benign orbital tumors with bone destructions in children.

Case/Age(yr)/Sex/SideDiagnosis	Symptoms/Duration(wk)	Physical findings	CT findings	Intervention	Follow-up	Outcome
**1**/3/M/REosinophilic granuloma	Eyelid swelling/5;pain	Upper eyelidedema; erythema;palpable mass	Low-density lesion;extensive destruction of anterolateral frontal bone	Incisional biopsy	20 m	No other focino recurrencenormal vision
**2**/3/F/LEosinophilic granuloma	Eyelid swelling/4;ptosis	Upper eyelid edema;ptosis;palpable mass	Superior-temporal mass;bone destruction of anterior frontal bone	Incisional biopsy10 Gy radio	18 m	No other focino recurrencenormal vision
**3**/7/M/REosinophilic granuloma	Eyelid swelling/1;pain;proptosisdecreased vision	Upper eyelid edema;erythema;tenderness	Lateral soft tissue mass;bone destructionof lateral wall	Subtotal curettage10 Gy radio	60 m	No other focino recurrencenormal vision
**4**/3/M/REosinophilic granuloma	Eyelid swelling/3;pain	Upper eyelid edema;erythema;tenderness	Superior soft tissue mass;bone destruction of anterior frontal bone	Subtotal curettagesystemiccorticosteroid	36 m	No other focino recurrencenormal vision
**5**/5/F/LEosinophilic granuloma	Eyelid swelling/4;proptosisdecreased vision	Lower eyelid edema;erythema;tenderness;palpable mass	Inferior-temporal mass;bone destruction of lateral and lower wall	Subtotal curettagesystemic corticosteroid	32 m	No other focino recurrencenormal vision
**6**/6/M/LEosinophilic granuloma	Eyelid swelling/5;decreased vision	Upper eyelid edema;palpable mass	Superior-temporal mass;extensive destruction of anterolateral frontal bone	Subtotal curettagesystemic corticosteroid	24 m	No other focino recurrencenormal vision
**7**/3/F/LLeiomyoma	Lateral orbital lump/4	Palpable mass	Lateral soft tissue mass;bone destruction of lateral wall	Subtotal curettage	36 m	No recurrencenormal vision
**8**/3/M/RIntraosseous hemangioma	Lower orbital lump/4	Lower eyelid edema;palpable mass	Inferior soft tissue mass;bone destruction of lower orbital wall	Subtotal curettage	36 m	No recurrencenormal vision

**Figure 1:**
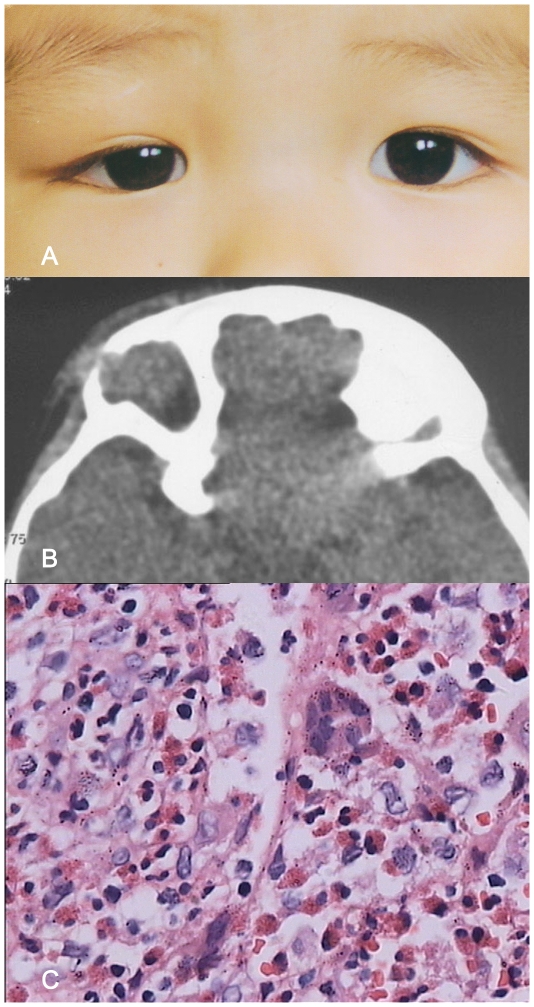
A–C: Patient with orbital eosinophilic granuloma (case4). Figure 1A: Clinical appearance of fullness of the upper eyelid of the right eye. Figure 1B: Computed tomography (CT) shows erosion of an intraorbital soft tissue mass through anterior and posterior cortex of frontal bone, similar to malignant tumors. Figure 1C: The tumor tissues comprised pathologic Langerhans cells, eosinophils, scat-tered lymphocytes, plasma cells, and multinucleated giant cells (magnification ×400; hematoxylin-eosin stain).

The histopathological examinations of specimens were suggestive of eosinophilic granuloma in 6 cases, and leiomyoma and primary orbital intraosseous hemangioma in 1 case respectively. Light microscopic examination of formalin-fixed specimens showed fairly uniform findings in patients with eosinophilic granuloma. The tumor tissues were comprised of pathologic Langerhans cells, eosinophils, scattered lymphocytes, plasma cells, and multinucleated giant cells. Immunohistochemical stainings for CD68, vimentin and S-100 were strongly positive in all 6 cases. In patient with leiomyoma, the histopathologic examination showed that the tumor was composed of spindle-shaped, benign-appearing cells organized in fascicles or loosely arranged in a myxoid stroma. In immunohistochemical staining, the specimen was positive for vimentin and alpha-smooth muscle actin, negative for S-100. The histopathologic finding of primary orbital intraosseous hemangioma was straightforward, consisting of the thin-walled blood vessels which are closely clustered and separated by normal bony tissue (Figure 1–3). After pathologic diagnosis, all patients with eosinophilic granuloma were evaluated by pediatric oncologists for systemic involvement. Every patient underwent a complete physical examination, routine laboratory testing, and radiographic skeletal survey. Other evaluation varied and included radionuclide bone scanning, abdominal and pelvic CT examination, blood chemistry and bone marrow aspiration. There was no evidence for multifocal Langerhans cell histiocytosis (LCH) in any case.

**Figure 2 pone-0032111-g002:**
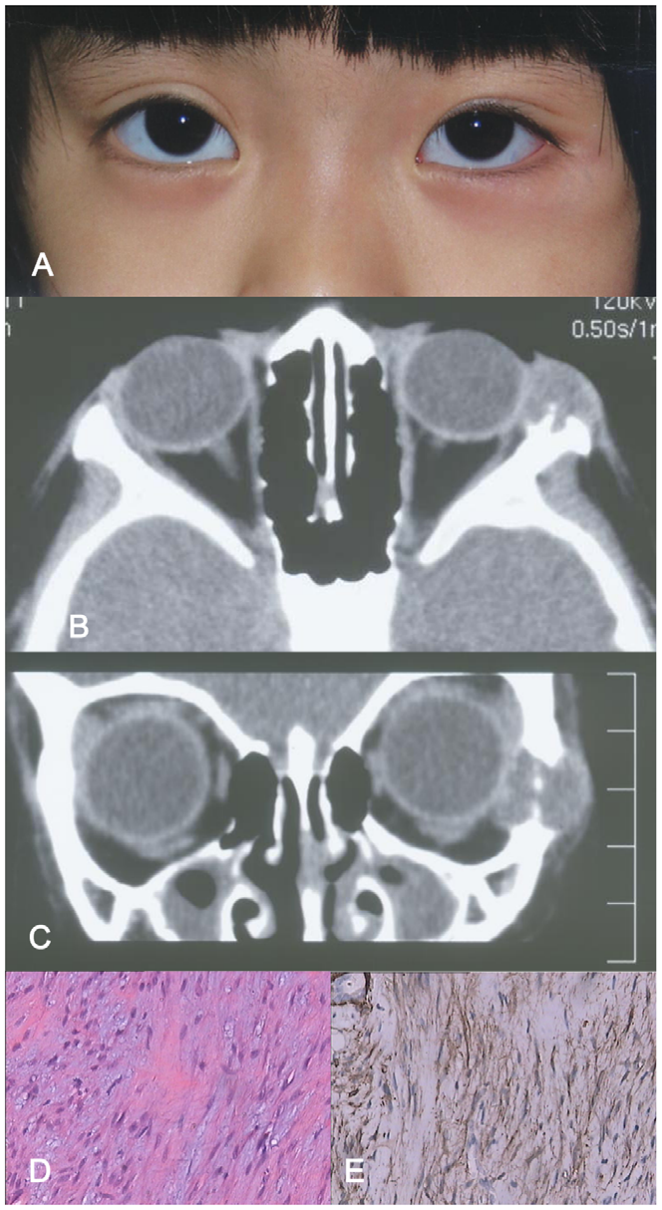
A–E: Patient with orbital leiomyoma(case7). Figure 2A: Clinical appearance of a hard, un-movable, well-marginated mass measuring 15 mm×10 mm in the left temporal periorbital area. Figure 2B, C: Computed tomography (Axial, Figure 2B; Coronal, Figure 2C) revealed a 22 mm×13 mm well-defined soft tissue mass. There was marked destruction of the lateral orbital wall. Figure 2D: The histopathologic examination showed that the tumor composed of spindle-shaped, benign-appearing cells organized in fascicles or loosely arranged in a myxoid stroma (magnification ×200; hematoxylin-eosin stain). Figure 2E: In immunohistochemical staining, the specimen was positive for alpha-smooth muscle actin (magnification ×200).

**Figure 3:**
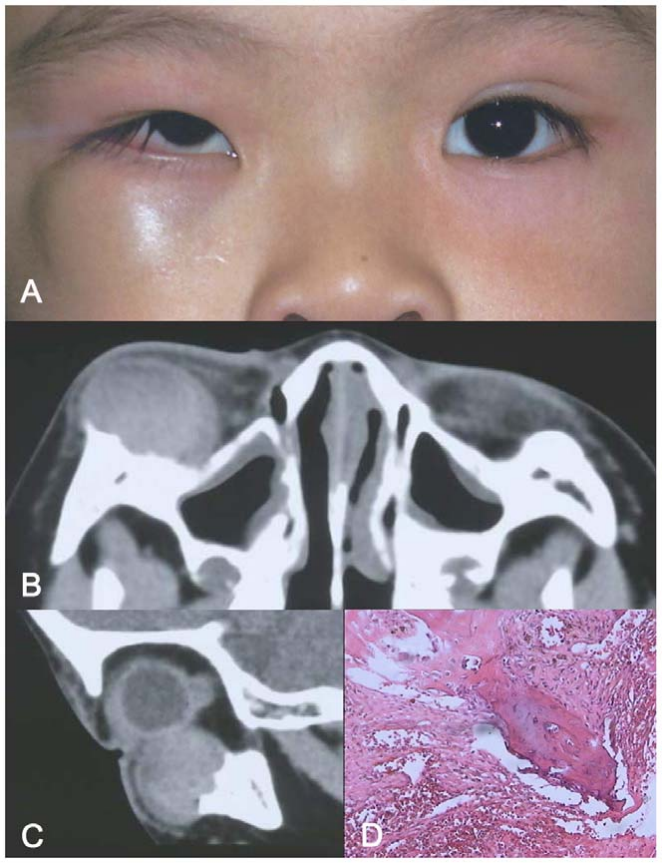
A–D: Patient with primary orbital intraosseous hemangioma complicated with hematoma (case8). Figure 3A: Clinical appearance of lower eyelid mass with obvious upward displacement of the right eye. Figure 3B, C: Computed tomography scan (Axial, Figure 3B; Sagittal, Figure 3C) disclosed a smoothly outlined homogeneous soft tissue mass in the inferior-anterior part of the right orbit, with remarkable bone destruction of the lower orbital rim. Figure 3D: The histopathologic finding of primary orbital intraosseous hemangioma, consisting of the thin-walled blood vessels which are closely clustered and separated by normal bony tissue (magnification ×200; hematoxylin-eosin stain).

Following definitive diagnosis, in six patients with eosinophilic granuloma, two patient were treated with fractionated external beam radiation to total doses of 10 Gy, three patients received systemic corticosteroid and one patients was periodically observed only. Authors usually choose the course of systemic corticosteroids as follows: Starting drug was dexamethasone 0.2 mg/kg per day for 3 days. Then they used oral prednisone 1 mg/kg per day for two weeks, and tapered slowly for two to three months. They did not use local intralesional corticosteroids for their patients. There was no postoperative therapeutic intervention in the two patients with leiomyoma and primary orbital intraosseous hemangioma. To authors' surprise, all eight patients were doing quite well after treatment. None of the 6 patients with eosinophilic granuloma had local recurrence, other foci of LCH, or other serious illness. The 2 patients with leiomyoma and primary orbital intraosseous hemangioma had no recurrence too. All 8 patients regained normal vision after a mean follow-up time of 32.8 months (range 18 months to 60 months).

## Discussion

Both benign and malignant masses of the orbit can have bone destruction [4]. Rarely, even orbital cavernous hemangioma may have bone erosion [5]. However, the benign lesions with bone destruction usually have definite clinical features and imaging appearances which may help clinician to differentiate them from maligancies. The dermoid cysts usually contain lipid and most often lie near the zygomaticofrontal suture, with an indolent-appearing erosion of bone. Fibrous dysplasia is a mass having a characteristic of ground-glass appearance, whereas juvenile ossifying fibroma is likely to produce a mixed lytic and sclerotic lesion and focal osseous enlargement [1]. Authors found several pediatric patients with patholoigcally confirmed benign lesions of the orbit who revealed remarkable bone destruction at imaging evaluation, really similar to malignant tumors.

Eosinophilic granuloma of the orbit is a subtype of Langerhans cell histiocytosis (LCH), an idiopathic reticuloendothelial proliferative disorder with clonal proliferative Langerhans cells. LCH embraces three main clinical subtypes (or syndromes): Unifocal eosinophilic granuloma disease, multifocal unisystem disease (Hand–Schüller–Christian syndrome) and multifocal multisystem disease (Letterer–Siwe syndrome). The isolated eosinophilic granuloma of the orbit is uncommon, with onset in the first or second decade, but predominantly in the 2–5 years age group. Males are affected twice as frequently as females [6]. Symptoms include rapidly progressive upper eyelid edema and erythema, bone pain, and tenderness. The process usually involves the bones of the lateral orbital roof; it produces lytic destruction of bone with a sclerotic rim and a large intraorbital soft-tissue mass. Computed tomography (CT) shows extensive frontal bone destruction, similar to malignant tumors. The MRI appearance of orbital eosinophilic granuloma usually was an isointensity mass on T1-weighted images and a high-intensity mass on T2 weighted images. Gd-DTPA clearly demonstrated the tumor extension. However, it really suggests a benign and self-limiting problem and requires biopsy and curettage only or combines with local or systemic corticosteroids [6], [7], [8]. Because the Langerhans cells produced cytokines IL-1 and PGE2, the disproportionate bone destruction appeared. As the major osteoclast-activating factor and a potent inhibitor of bone formation, IL-1 also interfered with collagen synthesis [9], [10]. It was reported in vitro bone resorption resulted from PGE2 [11]. It seems that pathologic Langerhans cells lead to osteolysis through the elaboration of PGE2 and IL-1 [6]. The definitive diagnosis of orbital eosinophilic granuloma is in view of identification of Langerhans cells, varying numbers of eosinophils, mononuclear, multinucleated histiocytes, neutrophils and small lymphocytes [6], [7], [8]. All patients need systemic investigation to eliminate involvement of other organs.

Leiomyoma is a benign smooth muscle tumor that is most commonly encountered in the uterus, skin, and gastrointestinal tracts [12], [13]. Leiomyomas of the orbit are very rare. To our knowledge, bone destruction is an unreported presentation of an orbital leiomyoma. The tumor is presumably derived from smooth muscle cells of vessel walls posteriorly and from the capsulopalpebral or Müller muscle anteriorly [12]. Arat et al recently reviewed 26 cases of orbital leiomyoma [13]. The age of the patients ranged from 9 years to 57 years (mean 30 years). Review of previously reported cases showed a male predilection (73%) [12], [13], [14]. The most common clinical presentation is a painless, slowly progressing proptosis over several months or years, without inflammation or pain that can be located anywhere in the orbit. Leiomyomas located in the anterior orbit may present with progressive painless swelling of the eyelids. The tumor can disturb extraocular motility in some cases. Computed tomography (CT) and MRI usually demonstrate a well-circumscribed orbital tumor. On MRI, orbital leiomyoma was isointense to the extraocular muscle on T1-weighted images and hyperintense on T2-weighted images, with moderate contrast enhancement. However, there are no specific characteristics which are helpful to radiologist for excluding other benign orbital lesions [12], [13], [14], [15], [16]. Therefore complete excision often has been chosen as the therapy [14]. Intimate follow-up is necessary, if without complete excision of mass. The tumor is resistant to radiation which is not suggested in orbital tumor [13]. Although it grew relatively fast and demonstrated remarkable bone destruction, the tumor of our case was deemed benign because it grew without malignant features (such as high mitotic count and significant cellular atypia). In immunohistochemical staining, the specimen was positive for vimentin and alpha-smooth muscle actin, negative for S-100. The post-operative course was uneventful. At the present time, 3 years after surgery, the patient is currently doing well, without evidence of recurrent disease, confirmed the diagnosis of leiomyoma, not leiomyosarcoma. Erosion of adjacent bone due to secondary compression phenomenon of vascular leiomyoma in other organs has rarely been reported in the literature [17], [18]. The mechanism is worth of further investigation.

Primary intraosseous hemangiomas are rare, usually solitary, benign, slow-growing neoplasms, with more than 50% being found in the vertebra or skull. Primary orbital intraosseous hemangiomas are extremely rare, only 45 cases were reported in the English literature to date [19]. The pathogenesis of these lesions is unknown. They are typically found in adults and occur more frequently in females than males with a ratio of 3 to 1. The two histologic types (cavernous and capillary) have similar imaging findings and are often differentiated by their histopathologic appearances. Plain radiographs demonstrates osteolytic lesion. Heterogeneous internal structures with honeycomb pattern and well-defined margins appear in CT scans. A sunburst of radiating trabecula with or without a thin peripheral sclerotic rim is the characteristic radiographic pattern [20], [21]. Patients may have signs with an asymptomatic lump, proptosis, diplopia, optic atrophy, and ptosis. Clinical differential diagnosis involves eosinophilic granuloma, fibrous dysplasia, osteoma, meningioma, multiple myeloma, osteosarcoma and metastatic disease. Patient may has obvious lesion without any symptoms. Otherwise, the necessary therapy is complete excision, but partial excision may lead to good result [21]. Authors' case has some unusual clinical features, such as the rapid onset of symptoms, complicated with a hematoma in the anterior orbit, in a patient with hemophilia, age at presentation, capillary type of hemangioma, only occurring at the orbital rim. The lesion exhibited remarkable bone destruction with round soft tissue mass on CT scan, mimicking the characteristics of a malignant lesion, so that the lesion was presumed to be orbital rhabdomyosarcoma pre-surgically. Follow-up examination performed 3 years after discharge revealed no recurrence of the tumor, and he has remained symptom free.

The differential diagnosis of benign orbital tumors with prominent bone destruction in children that simulate malignant tumors includes myofibroma, solitary fibrous tumors, haemangiopericytoma and so on. Orbit myofibroma typically occurs in childhood and presents as a firm, painless, well-circumscribed mass. Intraosseous occurance has been reported [22], [23]. Although histologically benign, lesions can be locally aggressive and demonstrate rapid growth and irregular osseous destruction [22]. Treatment consists of complete surgical excision. Histologically, myofibromas demonstrate a nodular arrangement of whorled, interlacing bundles of myofibroblasts. These cells blend into a central zone composed of less differentiated polygonal cells arranged around vascular channels [22]. Myofibroma is immunohistochemically positive for vimentin and smooth muscle actin and negative for desmin, CD34, S100 [22], [23], [24]. The solitary fibrous tumor (SFT) is an uncommon spindle-cell neoplasms of orbit and usually shows a slow-growing, painless extraconal lesion. It occurs over a wide age range, including children. CT and MRI usually reveal round to oval, well-circumscribed, contrast-enhancing lesions that may or may not cause bony erosion. Cytologic atypia, increased mitotic activity, and increased prognostic marker reactivity can identify histologically borderline or low-grade malignant ocular SFT [25]. Treatment of SFT includes complete surgical removal. Tumor cells are described as spindle shaped with scant cytoplasm and indistinct nucleoli. The tumor matrix contains a distinctive thick “ropey” type of collagen between the randomly oriented tumor cells. Immunohistochemically, these neoplasms are consistently immunoreactive for vimentin, CD34 and CD99, but negative for desmin and actin [26], [27]. Hemangiopericytomas(HPCs) are uncommon vascular tumors composed of an abnormal proliferation of pericytes. They usually occur in adults, but it has been noted in children [24], [26]. The major clinical features of orbital HPCs are proptosis and either extraconal or intraconal mass effect, but predominantly in the superior orbit, with bone erosion sometimes. On CT and MRI imaging, these tumors tend to present as well-defined masses with remarkable homogeneous enhancement. They have an unpredictable pattern of behavior and best handled by careful local excision [26], [28]. Under the microscope, HPCs are remarkable for their characteristic thin-walled, branching or “staghorn” blood vessels, with closely packed, randomly oriented cells and irregular, carrot-shaped nuclei. Mitotic activity and nuclear atypia are considered predictive of aggressive behavior. HPCs are diffusely immunoreactive for vimentin and CD34, but negative for S-100 protein [26]. Recently some authors proposed that orbital HPC can justifiably be redesignated as orbital SFT; they have a spectrum of overlapping morphologic and immunophenotypic findings suggestive of the previously subcategorized diagnoses [27], [28], [29].

In summary, some benign orbital tumors, including isolated eosinophilic granuloma, leiomyoma and primary orbital intraosseous hemangioma may show remarkable bone destruction in children. Therefore, we should pay attention to these rare orbital benign tumors in the differentiation of tumors with bone destruction. The differential diagnosis includes myofibroma, solitary fibrous tumors, haemangiopericytoma and so on.
